# ESE3-positive PSCs drive pancreatic cancer fibrosis, chemoresistance and poor prognosis via tumour–stromal IL-1β/NF–κB/ESE3 signalling axis

**DOI:** 10.1038/s41416-022-01927-y

**Published:** 2022-08-19

**Authors:** Tiansuo Zhao, Di Xiao, Fanjie Jin, Xugang Sun, Jie Yu, Hongwei Wang, Jing Liu, Wenrun Cai, Chongbiao Huang, Xiuchao Wang, Song Gao, Zhe Liu, Shengyu Yang, Chuntao Gao, Jihui Hao

**Affiliations:** 1 Department of Pancreatic Cancer, Tianjin Medical University Cancer Institute and Hospital, National Clinical Research Center for Cancer; Key Laboratory of Cancer Prevention and Therapy, Tianjin’s Clinical Research Center for Cancer, Tianjin, PR China; 2 Hepatopancreatobiliary Surgery Department, First Hospital of Shanxi Medical University, Taiyuan, PR China; 3 Department of Immunology, Biochemistry and Molecular Biology, Tianjin Medical University, Tianjin, PR China; 4 Department of Cellular and Molecular Physiology, Penn State College of Medicine, Hershey, PA USA

**Keywords:** Extracellular signalling molecules, Pancreatic cancer

## Abstract

**Background:**

Desmoplastic stroma, a feature of pancreatic ductal adenocarcinoma (PDAC), contains abundant activated pancreatic stellate cells (PSCs). How PSCs promote PDAC progression remains incompletely understood.

**Methods:**

Effect of epithelium-specific E-twenty six factor 3 (ESE3)-positive PSCs on PDAC fibrosis and chemoresistance was examined by western blot, RT-PCR, immunofluorescence, flow cytometry assay, chromatin immunoprecipitation, luciferase assay, immunohistochemistry and subcutaneous pancreatic cancer mouse model.

**Results:**

ESE3 expression increased in PSCs in PDAC tissues compared with those in normal PSCs. Clinical data showed that ESE3 upregulation in PSCs was positively correlated with tumour size, pTNM stage, CA19-9, carcinoembryonic antigen and serum CA242 level. ESE3 overexpression in PSCs was an independent negative prognostic factor for disease-free survival and overall survival amongst patients with PDAC. Mechanistically, the conditional medium from the loss and gain of ESE3-expressing PSCs influenced PDAC chemoresistance and tumour growth. ESE3 directly induced the transcription of α-SMA, collagen-I and IL-1β by binding to ESE3-binding sites on their promoters to activate PSCs. IL-1β upregulated ESE3 in PSCs through NF-κB activation, and ESE3 was required for PSC activation by tumour cell-derived IL-1β.

**Conclusion:**

Inhibiting the IL-1β/ESE3 (PSCs)/IL-1β-positive feedback loop is a promising therapeutic strategy to reduce tumour fibrosis and increase chemotherapeutic efficacy in PDAC.

## Background

Extensive desmoplasia, which is also referred to as a seriously fibrotic reaction, is a prominent feature of pancreatic ductal adenocarcinoma (PDAC) [1]. The desmoplastic stroma contains abundant activated pancreatic stellate cells (PSCs) and extracellular matrix (ECM) [2]. PSCs and ECM components form the fibrotic tumour microenvironment (TME), which influences the survival and migration of PDAC cells [3]. Furthermore, the interactions of cancer and stromal cells (PSCs, immune cells, etc.) with the ECM form a physical barrier that limits the delivery of chemotherapeutic agents and causes chemoresistance [4–6]. Therefore, understanding how PSCs promote chemoresistance and poor prognosis of PDAC should be urgent.

The stromal inflammatory cells (macrophages, neutrophils and mast cells) surrounding tumours cross-talk with PDAC cells and create an immunosuppressive environment that is resistant to chemotherapy [7]. Immunosuppressive inflammatory cells, including tumour-associated macrophages, myeloid-derived suppressor cells and regulatory T cells, play a crucial role in PDAC progression and chemoresistance [6, 8, 9]. Chemotherapy-derived inflammatory responses accelerate the formation of immunosuppressive myeloid cells in the tissue microenvironment of PDAC [10]. Proinflammatory cytokines, including IL-1β and IL-6, are associated with the efficacy of chemotherapy in patients with PDAC. Serum IL-1β and IL-6 levels influence the efficacy of gemcitabine (Gem) therapy in PDAC [11]. Adipocyte- and PSC-secreted IL-1β recruits tumour-associated neutrophils, activates PSCs and promotes pancreatic cancer progression and chemoresistance [4]. IL-1β derived from tumour and stromal cells forms an IL-1β–IRAK4 feedforward signal that drives tumour fibrosis, chemoresistance and poor prognosis in PDAC [12, 13]. In various chemoresistant PDAC cells, constitutive NF-κB activation causes the autocrine action of IL-1β [14, 15].

Therefore, IL-1β in the TME can be derived from immune cells, PSCs and PDAC cells, and the disruption of IL-1β/PSC signals can increase chemotherapeutic efficacy in PDAC.

Epithelium-specific E-twenty-six factor 3 (ESE3) belongs to an E-twenty-six (ETS) transcription factor subfamily characterised by epithelium-specific expression (ESE) [16]. All proteins contain a conserved DNA-binding motif, which binds to the ETS-binding site (EBS, GGAA/T) in the promoter and the regulatory sequences of target genes [17]. *ESE3,* as a tumour suppressor gene, binds directly to target genes to regulate epithelial cell differentiation, metastasis, stem-like features, epithelial-to-mesenchymal transition (EMT), tumourigenesis and tumour progression in pancreatic cancer and prostate cancer [18–21]. However, *ESE3* as an oncogene promotes thyroid tumourigenesis [22], ovarian cancer [23] and gastric cancer [24] progression.

Silverman et al. reported that IL-1β or TNF-α can induce ESE3 mRNA and protein expression in human bronchial smooth muscle cells and fibroblasts through the MAPK pathway [25]. Up to now, the expression, function and molecular mechanisms underlying ESE3 in PSCs are still unknown.

In this study, we discovered that ESE3 overexpression in PSCs is crucial for IL-1β-induced PSC activation and PDAC chemoresistance. Mechanistically, ESE3 activates PSCs by directly upregulating the transcription of α-SMA, collagen-I and IL-1β. IL-1β promotes ESE3 overexpression in PSCs through NF-κB activation. Importantly, the stromal expression of ESE3 is an independent predictor of PDAC progression and survival. Our findings revealed a novel IL-1β/ESE3/IL-1β feedback signalling loop in PSC chemoresistance and PDAC fibrosis and progression.

## Methods

### Immunohistochemistry (IHC) and Masson’s trichrome staining

PDAC samples were obtained from 107 patients (aged 26–78 years) undergoing surgical resection with a histological diagnosis of PDAC at Tianjin Medical University Cancer Institute and Hospital. The patients’ histopathological and clinical characteristics are shown in Supplementary Table S1. IHC for ESE3 was performed on the samples using a diaminobenzidine substrate kit (ZSGB-BIO, Beijing, China). Immunoreactivity was semi-quantitatively scored according to the estimated percentage of positive tumour cells as previously described [18]. Masson’s trichrome staining using a commercial kit (Solarbio, G1346).

### Immunofluorescence (IF)

Human primary PSCs (hpPSCs) were seeded onto glass slides for different treatments to assess ESE3 and α-SMA distribution. The cells were then washed once with PBS and fixed with 4% paraformaldehyde in PBS for 15 min, permeabilised with 0.1% Triton X-100 in PBS for 30 min at room temperature and blocked for 1 h with 3% BSA in PBS. Then, the cells were stained with anti-ESE3 and α-SMA antibodies (1:200 dilution) overnight at 4 °C. The cells were mounted with diamidinophenylindole (DAPI) Fluoromount-G media with DAPI nuclear stain (Southern Biotech). The slides were viewed with a Keyence microscope.

### PDAC cell culture and human and mouse primary PSC isolation and culture

Human PDAC cell lines MIA-PaCa-2, BxPC-3, SW1990 and HEK293 were obtained from the cell bank of the Type Culture Collection of Chinese Academy of Sciences (Shanghai, China). The human PDAC cell line L3.7 was a gift from Prof. Keping Xie (MD Anderson Cancer Centre, Houston, TX). All the cell lines were authenticated in 2018 through the short tandem repeat analysis method. The cells were grown at 37 °C in a humidified atmosphere with 95% air and 5% CO_2_ using Dulbecco’s modified Eagle medium (DMEM) with 10% foetal bovine serum (FBS).

The hpPSCs were isolated by the outgrowth method. The PDAC surgical specimens obtained from the patients at Tianjin Medical University Cancer Institute and Hospital were cut into small pieces with a sharp scalpel. The PDAC tissue blocks were attached to the bottom of the 10-cm dish and carefully added with 3 mL of DMEM1/2 medium containing 20% FBS, 100 U/mL penicillin and 100 μg/mL streptomycin around the blocks. PSCs were identified by IF with α-SMA staining [26].

Mouse primary PSCs (mpPSCs) were isolated from the pancreas of healthy C57BL/6 mice (3 months old) by collagenase digestion followed by Nycodenz® (Nycomed, Oslo, Norway) density-gradient centrifugation [27]. Afterwards, the PSCs were cultured in DMEM1/2 medium containing 20% FBS, 100 U/mL penicillin and 100 μg/mL streptomycin.

The human PSCs were immortalised by transfection with SV40 large T antigen and human telomerase (named: immortal human PSCs (ihPSCs)) [28].

### siRNA duplexes, plasmid constructs, transient transfection, stable transfection and dual-luciferase assay

siRNAs against ESE3 and NF-κB were designed and synthesised from GenePharma (Shanghai, China; Supplementary Table S2).

For ESE3-knockdown in PSCs, shRNA sequences were designed by Sigma-Aldrich shRNA designer (Supplementary Table S2). The recommended sequences for ESE3 genes were synthesised and cloned into the pLVi-shRNA puro Vectors (Biosettia).

Human ESE3 cDNA was cloned into the pCDH plasmid expression vector. ESE3 overexpression in PSCs and lentivirus-mediated plasmid was performed using the pCDH-cDNA system (Biosettia) following the manufacturer’s instructions. Lentivirus-encoding DNAs were packaged as previously described [29]. The infected cells were selected with puromycin (1 μg/mL) for 1 day.

The genomic DNA fragments of human ESE3, α-SMA, collagen-I and IL-1β genes, spanning from +1 to −2000 relative to the transcription initiation sites, were generated by polymerase chain reaction (PCR) and inserted into pGL3-Basic vectors. All constructs were sequenced to confirm their identity. Luciferase activity was measured using the Dual-luciferase Reporter Assay System (Promega) according to the manufacturer’s instructions. The transfected cells were collected for cell migration analysis, Western blot analysis, reverse-transcription polymerase chain reaction (RT-PCR), apoptosis and cell cycle assays.

### Western blot analysis

Whole-cell extracts were prepared by lysing the cells with radioimmunoprecipitation lysis buffer supplemented with a proteinase inhibitor cocktail (Sigma). The nuclear and cytoplasmic proteins of PSCs were extracted according to the instructions of the nuclear–cytoplasm extraction reagents (Thermo Fisher Scientific, USA, Catalogue number: 78835). Protein lysates (20 μg) were separated by sodium dodecyl sulphate–polyacrylamide gel electrophoresis, and the target proteins were detected by western blot analysis with antibodies (Supplementary Table S2). Specific proteins were visualised using an enhanced chemiluminescence detection reagent (Pierce).

### RT-PCR

Total RNA was isolated from the transfected cells with TRIzol Reagent (Invitrogen) and used for first-strand cDNA synthesis using the First-strand Synthesis System for RT-PCR (Takara). The PCR primers used are indicated in Supplementary Table S2.

### PDAC–PSC co-culture, apoptosis and cell cycle assays

PDAC cells were indirectly co-cultured with PSCs. The supernatants of transfected PSCs were collected and used as the conditional medium (CM), and the PDAC cell lines were cultured with the CM.

The PDAC cells (2 × 10^5^) resuspended in DMEM containing 10% FBS were seeded in the lower chamber, and the transfected PSCs (2 × 10^5^) resuspended in 500 μL of medium containing 10% FBS were placed in the upper chamber (Transwell, Corning, USA). Then, the PDAC cell lines were treated with Gem. Apoptosis rate and cell cycle distribution were analysed by flow cytometry. Gem was obtained from Tianjin Medical University Cancer Institute and Hospital (Gemzar, Eli Lilly and Company).

### Cell proliferation EdU assay

L3.7, MIA-PaCa-2 and BxPC-3 cell lines were placed in a 24-well plate (3000 cells/well) and cultured with the CM of transfected PSCs for 48 h. Click additive solution (0.5 mL) from the Click-iT EdU Alexa Fluor 488 Cell Proliferation Assay Kit (Molecular Probes, Invitrogen, USA) was added to each well. The cells were incubated at room temperature for 30 min in the dark. The click reaction solution was removed, and the cells were washed three times with PBS and incubated with DAPI for 10 min for nuclear staining. The slides were viewed on a Keyence microscope for image acquisition.

### 3D collagen gel-contraction assay

Collagen-I from rat tail tendon (Corning, USA) was added to DMEM containing 10% FBS and 0.1 M NaOH, and the medium was placed in a 24-well plate. The sh-vector/sh-ESE3 or pCDH-Vector/pCDH-ESE3 PSCs (8 × 10^4^) were added to the collagen solution, the cells were placed in an incubator (37 °C, 5% CO_2_) for 30 min to coagulate, and the coagulated collagen was separated from the plate wall. The collagen shrinkage of each component was observed after 72 h of culture. Collagen gel diameter was measured using ImageJ imaging software (National Institutes of Health [NIH], Bethesda, MD).

### Animal studies in a subcutaneous pancreatic cancer mouse model

Female 4-week-old nude nu/nu mice were maintained in a barrier facility on HEPA-filtered racks. For proliferation study, the cells were harvested by trypsinisation, washed in PBS and resuspended in a 1:1 solution of PBS/Matrigel. Then, 1 × 10^6^ L3.7 with 1 × 10^6^ pCDH-Vector or pCDH-ESE3 ihPSC was injected subcutaneously into the two flanks of six nude nu/nu mice. For the chemoresistance study, L3.7 with transfected ihPSC (Group 1: pCDH-Vector or pCDH-ESE3 ihPSC, Group 2: sh-Vector or sh-ESE3 ihPSC; 1:1) was subcutaneously injected into the nude mice, and Gem (15 mg/kg, twice a week) was administered after tumour formation at ~7 days. Primary tumours were measured in three dimensions (*a*, *b*, *c*), and volume was calculated as *abc* × 0.52. Some of the tumour samples were fixated by formalin, embedded with paraffin and analysed using Masson’s trichrome staining and IHC staining.

### Chromatin immunoprecipitation assay (ChIP)

ChIP assay was performed using a commercial kit (Merck-Millipore) according to the manufacturer’s instructions. The PCR primers are listed in Supplementary Table S2.

### Enzyme-linked immunosorbent assay (ELISA)

The CMs of the Gem-treated PDAC cell lines were collected and centrifuged at 1500 rpm for 5 min. IL-1β production in the supernatant was determined using an ELISA kit according to the manufacturer’s instructions (Proteintech, China; Catalogue number: KE00021).

### NF-κB p65 transcription factor assay

NF-κB p65 activity was determined by p65 binding ELISA (Abcam, ab133112). The Binding ELISA Kit was used according to the manufacturer’s protocol using nuclear lysates, and the values were normalised to microgram protein as determined by protein estimation (Abcam, ab113474).

### RNA sequencing

hpPSCs were treated with IL-1β-recombined protein and control for 24 h. RNA extraction and sequencing were performed by Biomarker Technologies (China). The total RNA of each sample was extracted from the cells using TRIzol reagent. After quality control, the cDNA library was constructed through NEBNext Ultra RNA Library Prep Kit for Illumina (NEB, E7530) and NEBNext Multiplex Oligos for Illumina (NEB, E7500). Finally, the cDNA libraries of the samples were sequenced using an Illumina HiSeq™ sequencing platform. After using reference genome-based beads mapping, gene expression was calculated using FPKM values (fragments per kilobase of exon per million fragments mapped) by the Cufflinks software.

Subsequently, the differentially expressed genes (DEGs) between the hpPSCs treated with IL-1β and the control were determined by DESeq2 and Q-value (FDR < 0.01, log2FC > 1). Based on the list of DEGs, Kyoto Encyclopaedia of Genes and Genomes pathways were assigned to acquire the related and activated pathways, and gene set enrichment analysis (GSEA) was performed using the GSEA software (V4.1.0) from Broad Institute (https://www.gsea-msigdb.org/gsea/index.jsp). Every sample had three duplicates for RNA sequencing, Principal component analysis (PCA) was performed to judge the comparability of the replicates and the differences amongst different conditions.

### Statistical analysis

Two independent sample t-test was used for the comparison between the two groups. ANOVA and post hoc Student–Newman–Keuls-q test were used for multiple comparisons. Chi-square test was used to correlate the expression level of ESE3 in PSCs with clinicopathological data. Pearson correlation analysis was used to analyse the correlation between ESE3 and Masson’s staining area in tissues. Kaplan–Meier method was used to estimate the overall survival (OS) or disease-free survival (DFS) in different ESE3 expression levels in PDAC. The log-rank method was used to compare the differences in OS and DFS. Cox proportional hazards regression model was used to assess the relationship between ESE3 expression levels in PSCs and OS/DFS. Each experiment was conducted independently for at least three times, and the data were presented as mean ±  standard deviation (SD). All tests presented were two-tailed, and *P* < 0.05 was considered statistically significant. Statistical analysis was performed using SPSS 20.0 and GraphPad Prism software.

## Results

### ESE3 was overexpressed in PSCs and associated with PDAC fibrosis

**Fig. 1:**
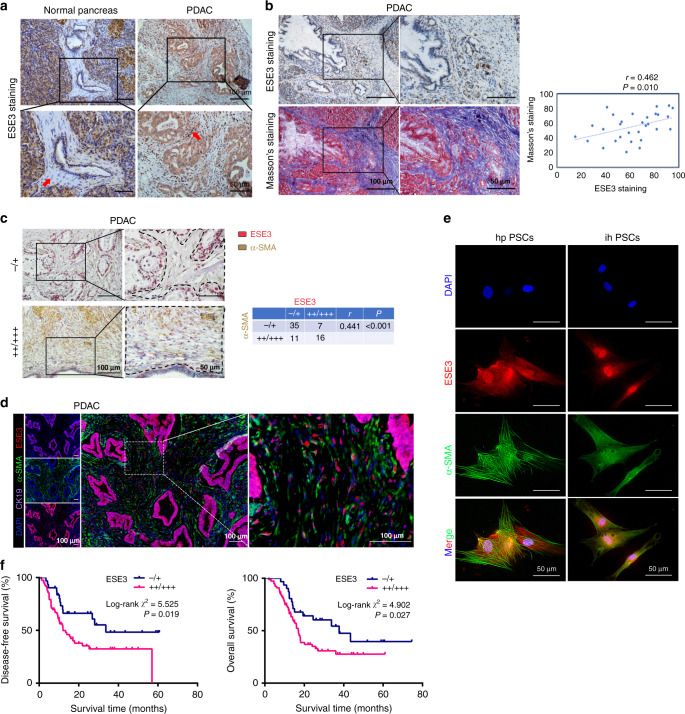
ESE3 was overexpressed in PSCs and associated with PDAC fibrosis. **a** IHC of ESE3 protein expression in PSCs and adjacent normal pancreatic tissues. **b** IHC and Masson’s trichrome staining in consecutive PDAC tissue sections. **c** Double immunohistochemical staining and correlation analysis between ESE3 protein expression and extent of stromal fibrosis (α-SMA, stromal marker) in PDAC samples. **d** Multiplex IF staining of ESE3 (red), α-SMA (green) and cytokeratin (CK-19, an epithelial marker, purple) in PDAC samples. **e** IF staining of ESE3 and α-SMA expression in hpPSCs and ihPSCs. **f** Association of ESE3 expression in PSCs with DFS (left) and OS (right) in PDAC.

Firstly, ESE3 expression in PDAC samples was examined by IHC staining. Interestingly, ESE3 was upregulated in PSCs in PDAC tissues compared with those in normal PSCs (Fig. 1a). Then, IHC and Masson’s trichrome staining in consecutive PDAC tissue sections demonstrated that ESE3 (PSCs) expression was associated with PDAC fibrosis (Fig. 1b, *P* = 0.010). ESE3 and α-SMA were tested by IHC to further verify the function of ESE3 (PSCs) in PDAC fibrosis. The data showed that ESE3 (PSCs) and α-SMA expression levels were associated with each other (Fig. 1c, *P* < 0.001).

IF staining of ESE3 in human PDAC tissues indicated that ESE3 protein was also expressed in PSCs, which was consistent with the IHC findings (Fig. 1d). Furthermore, IF assays were performed in hpPSCs and ihPSCs to verify ESE3 expression and location. ESE3 expression can be found in the cytoplasm and nucleus of PSCs, most ESE3 expression in the nucleus and less expression in the cytoplasm (Fig. 1e). Our data supported that ESE3 was overexpressed in PSCs and associated with PDAC fibrosis.

### ESE3 (PSC) was associated with decreased DFS and OS in PDAC patients

ESE3 IHC score was evaluated by two independent pathologists to explore the pathological importance of ESE3 (PSCs) in PDAC progression. The correlation between ESE3 (PSCs) and the clinicopathological features of PDAC was evaluated (Supplementary Table S3). ESE3 (PSCs) had no significant correlations with gender, age, differentiation, lymph node metastasis and histologic grade but was positively correlated with pTNM (*χ*^2^ = 6.343, *P* = 0.012), tumour size (*χ*^2^ = 6.405, *P* = 0.011), CA19-9 (*χ*^2^ = 4.845, *P* = 0.028), carcinoembryonic antigen (CEA; *χ*^2^ = 6.060, *P* = 0.014) and CA242 (*χ*^2^ = 4.046, *P* = 0.044) (Supplementary Table S3). Importantly, the Kaplan–Meier analysis of IHC data indicated that patients with PDAC who had moderate (++) or high (+++) ESE3 (PSCs) levels had significantly worse DFS and OS than those with negative (−) or low (+) ESE3 protein expression (*P* < 0.05; DFS: 14.00 and 33.70 months; OS: 17.00 and 33.73 months, respectively) (Fig. 1f). Univariate and multivariate analyses of the clinical follow-up data indicated that ESE3 expression in PSCs was an independent risk factor for OS and DFS (Supplementary Table S4). These data demonstrated that ESE3 (PSCs) was positively correlated with pTNM, tumour size, CA19-9, CEA and CA242 and negatively correlated with DFS and OS in patients with PDAC.

### ESE3-positive PSCs drove PDAC proliferation and chemoresistance in vitro and in vivo

**Fig. 2:**
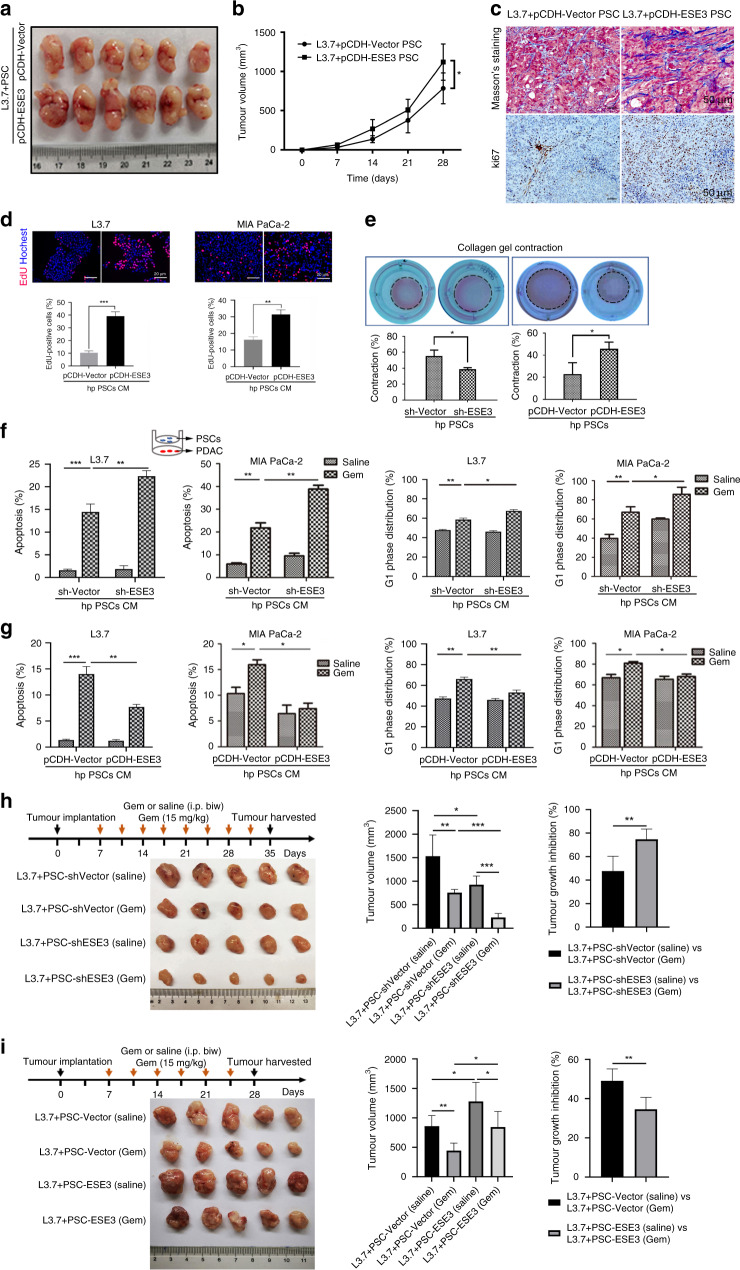
ESE3 (PSCs) drove PDAC proliferation and chemoresistance in vitro and in vivo. **a** Representative images of ihPSCs and PDAC cells (1:1) subcutaneously implanted into the nude mice (nu/nu). **b** Curves of tumour growth are expressed as mean ± SD with different groups. **c** Masson’s trichrome staining and Ki-67 staining analysis of the extent of stromal fibrosis and cell proliferation in mouse xenograft samples from different groups. **d** EdU experiment evaluation of the proliferation of L3.7 and MIA-PaCa-2 cells cultured with the CM of pCDH-ESE3/pCDH-Vector hpPSCs for 48 h. **e** Representative images from the 3D collagen gel contractility assay showing the contractility of sh-ESE3/sh-Vector hpPSCs (left) and pCDH-ESE3/pCDH-Vector hpPSCs (right) after 72 h. Flow cytometry was performed to analyse the apoptosis and G1-phase distribution of L3.7 and MIA-PaCa-2 cells indirectly co-cultured with sh-ESE3/sh-Vector hpPSCs (**f**) or pCDH-ESE3/pCDH-Vector hpPSCs (**g**) and then treated with Gem (2 μM) for 24 h. **h** L3.7+sh-Vector or sh-ESE3 ihPSCs (1:1) subcutaneously injected into nude mice treated with Gem or saline. Representative images, tumour volume and tumour growth inhibition are listed. **i** L3.7+pCDH-Vector or pCDH-ESE3 ihPSCs (1:1) subcutaneously injected into nude mice treated with Gem. Representative images, tumour volume and tumour growth inhibition are listed. *P < 0.05, **P < 0.01 and ***P < 0.001.

PSCs and L3.7 cells mixture (1:1) were subcutaneously injected into both flanks of nude nu/nu mice to evaluate whether ESE3 (PSCs) overexpression drives PDAC progression in vivo. Compared with the control group (pCDH-Vector ihPSC and L3.7 cell), the average tumour volume in the experiment group (pCDH-ESE3 ihPSC and L3.7 cell) was remarkably increased (Fig. 2a, b). Masson’s trichrome staining and IHC were used to verify the extent of fibrosis and ki67 expression in tumour cells and PSCs using the mouse xenograft samples. Masson’s trichrome staining and ki67 in tumour and PSCs were elevated remarkably in the experiment group (pCDH-ESE3 ihPSC and L3.7 cell) compared with the control group (pCDH-Vector ihPSC and L3.7 cell, Fig. 2c).

hpPSCs with ESE3 knockdown or overexpression were constructed (sh-ESE3/pCDH-ESE3 hpPSCs) to determine whether ESE3 (PSCs) plays a role in PDAC proliferation and chemoresistance. ESE3 expression level was confirmed by western blot (Supplementary Fig. S1). EdU assay demonstrated that the proliferation of the L3.7, MIA-PaCa-2 and BxPC-3 cell lines was induced obviously by the CM from pCDH-ESE3 hpPSCs compared with the CM from pCDH-Vector hpPSCs (Figs. 2d and Supplementary Fig. S2A). Collagen gel contractility assay showed that the contractility of sh-ESE3 hpPSCs decreased compared with that of sh-Vector hpPSCs, and the contractility of pCDH-ESE3 hpPSCs increased compared with that of pCDH-Vector hpPSCs (Fig. 2e). The results proved that ESE3 expression in PSCs promoted proliferation and fibrogenesis.

PDACs were indirectly co-cultured with sh-ESE3/sh-Vector or pCDH-ESE3/pCDH-Vector hpPSCs, and the effects of co-culture on PDAC cell apoptosis and cell cycle were analysed. Gem was used to induce PDAC (L3.7, MIA-PaCa-2 and BxPC-3) apoptosis and G1-phase cell cycle arrest. The apoptosis rate and G1-phase cell cycle arrest of the PDACs treated with Gem were induced after co-culture with sh-ESE3 hpPSCs compared with those co-cultured with sh-Vector hpPSCs (Fig. 2f and Supplementary Fig. S2B). Conversely, the apoptosis rate and G1-phase cell cycle arrest of the PDACs treated with Gem were reduced after co-culture with pCDH-ESE3 hpPSCs compared with those co-cultured with pCDH-Vector hpPSCs (Fig. 2g and Supplementary Fig. S2C).

Mouse xenograft models treated with Gem was established to further verify whether ESE3 affects PDAC chemoresistance. For the chemoresistance study, the nude mice were subcutaneously injected with L3.7+sh-Vector ihPSC or sh-ESE3 ihPSC (1:1) and then treated with Gem (15 mg/kg, twice a week) after tumour formation at 7 days. The results showed that the ESE3 knockdown group had increased sensitivity to Gem (tumour growth inhibition was 74.70% versus 47.71% in the sh-Vector group, *P* < 0.01; Fig. 2h). Furthermore, the nude mice were subcutaneously injected with L3.7+pCDH-Vector ihPSC or pCDH-ESE3 ihPSC (1:1) and then treated with Gem. The data showed that the ESE3 overexpression group had reduced sensitivity to Gem (tumour growth inhibition was 34.55% versus 49.06% in the pCDH-Vector group, *P* < 0.01; Fig. 2i).

Our data indicated that ESE3 (PSC) is crucial for PSCs to promote PDAC proliferation and chemoresistance in vivo and in vitro.

### ESE3 induced α-SMA, collagen-I and IL-1β expression in PSCs

**Fig. 3:**
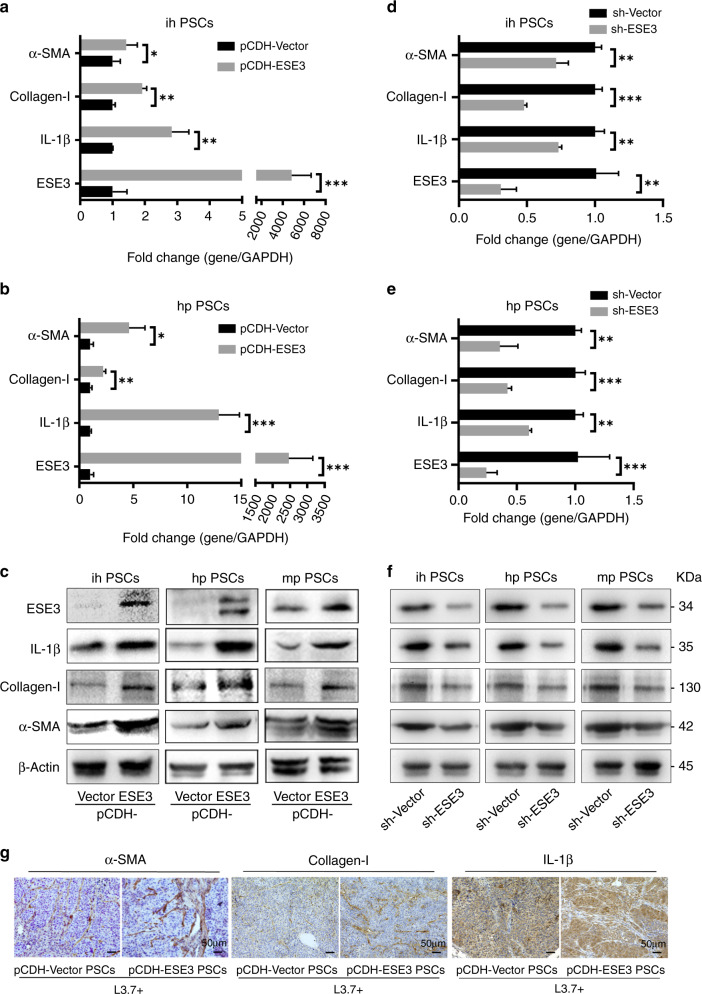
ESE3 induced α-SMA, collagen-I and IL-1β expression in PSCs. mRNA expression levels of α-SMA, collagen-I and IL-1β in ihPSCs (**a**) and hpPSCs (**b**) transfected with pCDH-ESE3 or pCDH-Vector plasmids. **c** Protein expression levels of α-SMA, collagen-I and IL-1β in ihPSCs, hpPSCs and mpPSCs transfected with pCDH-ESE3 or pCDH-Vector plasmids. mRNA expression levels of α-SMA, collagen-I and IL-1β in ihPSCs (**d**) and hpPSCs (**e**) transfected with sh-Vector or sh-ESE3 plasmids. **f** Protein expression levels of α-SMA, collagen-I and IL-1β in ihPSCs, hpPSCs and mpPSCs transfected with sh-Vector or sh-ESE3 plasmids. **g** IHC analysis of α-SMA, collagen-I and IL-1β expression levels in the mouse xenograft samples. *P < 0.05, **P < 0.01 and ***P < 0.001.

The elevated α-SMA expression, ECM protein (such as collagens) accumulation and inflammatory cytokine secretion in PSCs are markers of pancreatic fibrosis in chronic pancreatitis and pancreatic cancer [30]. The effect of ectopic ESE3 on the expression of α-SMA, collagen-I and IL-1β was examined to investigate the role of ESE3 in PSC activation. pCDH-ESE3 plasmid was transfected in ihPSCs, hpPSCs and mpPSCs. PCR and western blot demonstrated that the mRNA and protein levels of α-SMA, collagen-I and IL-1β were remarkably upregulated by ectopically expressed ESE3 (Fig. 3a–c). Next, we knocked down the ESE3 expression in PSCs by shESE3 plasmid and investigated the expression of α-SMA, collagen-I and IL-1β. PCR and western blot indicated that the mRNA and protein levels of α-SMA, collagen-I and IL-1β were remarkably downregulated after ESE3 knockdown (Fig. 3d–f).

IHC staining indicated that α-SMA, collagen-I and IL-1β expression levels in PSCs were elevated in the experiment group (pCDH-ESE3 ihPSCs and L3.7 cells) compared with the control group (pCDH-Vector ihPSCs and L3.7 cells) using the mouse xenograft samples (Fig. 3g).

The results suggested that ESE3 induced PSC activation in vitro and aggravated the fibrotic reaction in xenograft tumours.

### ESE3 directly bound to the promoter regions of α-SMA, collagen-I and IL-1β to upregulate their expression in PSCs

**Fig. 4:**
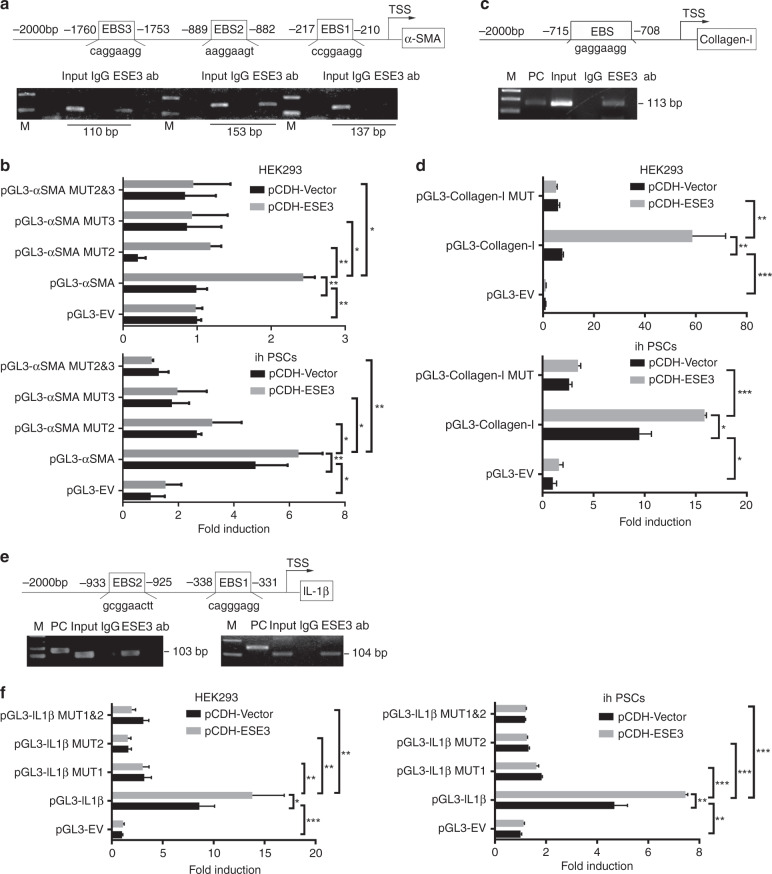
ESE3 directly bound to the promoter regions of α-SMA, collagen-I and IL-1β to upregulate their expression in PSCs. Schematic of the structures of the α-SMA (**a**), collagen-I (**c**) and IL-1β (**e**) gene promoters. Shown are the EBSs and their location (upper). ChIP analyses of ESE3 binding to the α-SMA (**a**), collagen-I (**c**) and IL-1β (**e**) promoters in ihPSCs (lower). Luciferase assay-based promoter activity analysis of the HEK293 and ihPSCs overexpressing ESE3 (pCDH-ESE3) and the control (pCDH-Vector) cells transfected with pGL3-α-SMA (**b**), pGL3-collagen-I (**d**), pGL3-IL-1β (**f**), pGL3-Empty Vector (pGL3-EV) and pGL3-Mutations (pGL3-MUT). The cells were subjected to dual-luciferase analysis 48 h after transfection. The results are expressed as fold induction relative to that in the corresponding cells transfected with the control vector after the normalisation of firefly luciferase activity according to *Renilla* luciferase activity. Data are expressed as means ± SD from three independent experiments. M means marker. *P < 0.05, **P < 0.01 and ***P < 0.001.

The promoter regions of α-SMA, collagen-I and IL-1β genes were surveyed, and EBSs (GGAA/T) were identified in their promoters to understand the molecular mechanism by which ESE3 activates PSCs (Fig. 4a, c, e, top). ChIP and luciferase analysis were performed. The fragments immunoprecipitated by anti-ESE3 antibody were detected in the chromatin fractions pulled down by an anti-ESE3 antibody (Fig. 4a, c, e, bottom). Different α-SMA, collagen-I and IL-1β luciferase promoter-reporter constructs were constructed and co-transfected with pCDH-Vector or pCDH-ESE3 plasmid into HEK293 cell and ihPSCs to determine whether the binding of ESE3 activates α-SMA, collagen-I and IL-1β promoters. Luciferase analysis showed that ESE3 overexpressed by the pCDH-ESE3 plasmid considerably increased α-SMA, collagen-I and IL-1β promoter activity in HEK293 cells and ihPSCs (Fig. 4b, d, f). These EBSs were mutated to determine whether the EBSs are required for ESE3 to transactivate the α-SMA, collagen-I and IL-1β promoters. As shown in Fig. 4b, d, f, the mutation of EBSs almost abolished the transactivation of the α-SMA, collagen-I and IL-1β promoters by ESE3. These data indicated that ESE3 directly binds to the promoters of α-SMA, collagen-I and IL-1β to induce the expression of these genes and activate PSCs.

### Tumour-secreted IL-1β induced nuclear ESE3 (PSCs) expression by activating NF-κB

**Fig. 5:**
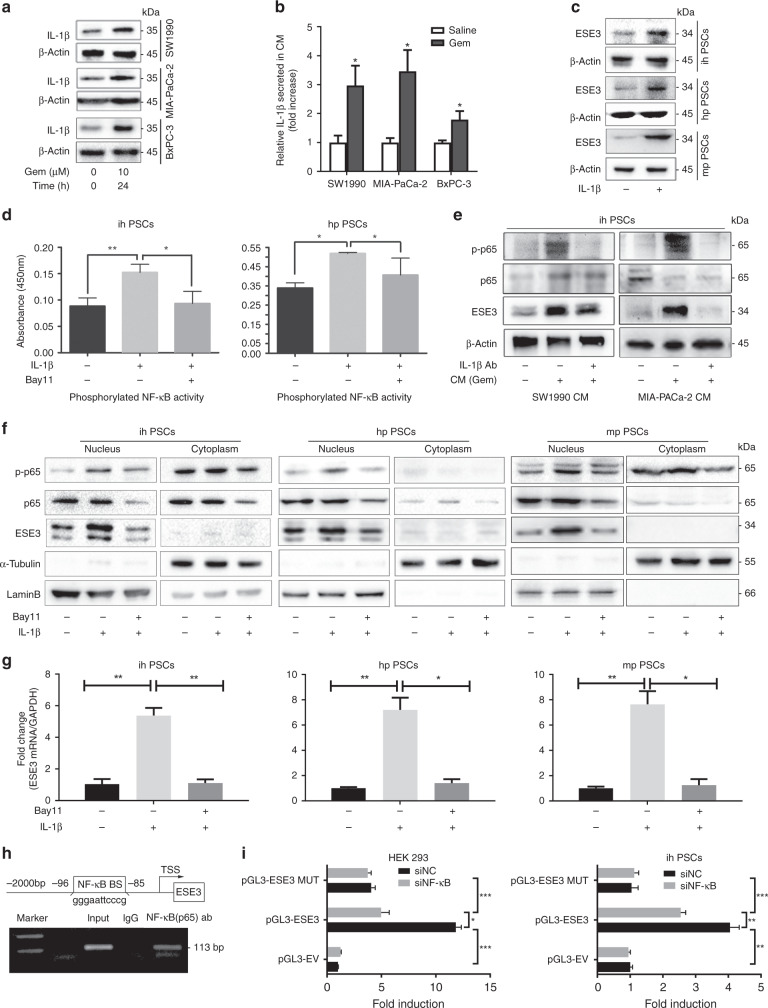
Tumour-secreted IL-1β induced nuclear ESE3 (PSCs) expression by activating NF-κB. **a** Western blot analysis of IL-1β expression in SW1990, MIA-PaCa-2 and BxPC-3 cell lines treated with Gem (10 μM) for 24 h. **b** ELISA of IL-1β secretion in the CMs of SW1990, MIA-PaCa-2 and BxPC-3 cell lines treated with Gem (10 μM) for 24 h. **c** Western blot analysis of ESE3 in ihPSCs, hpPSCs and mpPSCs treated with recombinant human IL-1β (100 ng/mL, 24 h). **d** Analysis of NF-κB (p65) transcriptional activities in ihPSCs and hpPSCs treated with recombinant human IL-1β (100 ng/mL, 24 h) and/or NF-κB (p65) inhibitor (Bay11, 8 μM; 12 h) by commercial kit. **e** Western blot analyses of p-p65 and ESE3 in ihPSCs cultured with CM (SW1990 and MIA-PaCa-2 cell lines treated with Gem) and/or IL-1β-neutralising antibody for 24 h. **f** Western blot analyses of p-p65 and ESE3 expression in nucleus and cytoplasm separation from the whole protein of PSCs treated with recombinant human IL-1β and/or Bay11. **g** RT-PCR analysis of ESE3 mRNA expression in ihPSCs, hpPSCs and mpPSCs treated with recombinant human IL-1β and/or Bay11. **h** Schematic of the structure of the *ESE3* gene promoter. Shown is one κB-binding site and its location (upper). ChIP analysis of NF-κB binding to the ESE3 promoter in ihPSCs (lower). **i** Luciferase assay-based promoter activity analysis of HEK293 (left) and ihPSCs (right) knockdown NF-κB (sip65) and control cells (siNC) transfected with pGL3-ESE3, pGL3-Empty Vector (pGL3-EV) and pGL3-Mutation (pGL3-MUT). The cells were subjected to dual-luciferase analysis 48 h after transfection. The results are expressed as fold induction relative to that in the corresponding cells transfected with the control vector after the normalisation of firefly luciferase activity according to *Renilla* luciferase activity. The data are expressed as means ± SD from three independent experiments. *P < 0.05, **P < 0.01 and ***P < 0.001.

IL-1β plays an important role in pancreatic fibrosis and PSC activation [31]. Previous studies demonstrated that IL-1β is produced by pancreatic cancer stroma [4, 32, 33]. Recent research found that tumour cell-derived IL-1β is essential for the establishment of pancreatic cancer desmoplasia [13]. We further tested IL-1β expression in PDAC cell lines treated with or without Gem. Western blot analysis indicated that tumour cell-derived IL-1β protein increased after Gem treatment (Fig. 5a). ELISA demonstrated a considerable increase in secreted IL-1β concentration in the CM of PDAC cell lines treated with Gem (Fig. 5b). Given the role of IL-1β in PSC activation, we examined the role of IL-1β in ESE3 (PSCs) expression by treating PSCs with recombined IL-1β or DMSO. IL-1β treatment was sufficient to induce ESE3 expression in PSCs (Fig. 5c).

RNA sequencing was performed to find the pathway activated by IL-1β stimulation and understand the mechanism by which IL-1β regulates ESE3. Pathway enrichment analysis showed that NF-κB signalling was remarkably activated in IL-1β-treated PSCs (Supplementary Fig. S3). Bay11 was used to block the NF-κB signal pathway to determine whether IL-1β induces ESE3 (PSCs) expression by activating NF-κB. NF-κB (p65) transcriptional activity in ihPSCs and hpPSCs treated with or without recombinant human IL-1β was further confirmed using p65 binding ELISA assays. IL-1β remarkably activated NF-κB (p65) transcriptional activity, which can be blocked by Bay11 (Fig. 5d).

The ihPSCs were treated with CM or non-Gem-treated CM to determine if tumour-derived CM induces ESE3 (PSCs) overexpression. The CM was collected from the SW1990 and MIA-PaCa-2 cell lines treated with Gem. Western blot indicated that ESE3 expression was upregulated in the ihPSCs treated with CM compared with those treated with non-Gem-treated CM (Fig. 5e). Moreover, CM-induced ESE3 (PSCs) overexpression can be blocked by IL-1β neutralising antibody (Fig. 5e). The nuclear p-p65 and ESE3 protein expression levels were also considerably upregulated in ihPSCs, hpPSCs and mpPSCs treated with recombinant IL-1β (Fig. 5f). The IL-1β-induced ESE3 overexpression and p65 phosphorylation at the protein and mRNA levels were inhibited by p65 inhibitor Bay11, suggesting that IL-1β induces ESE3 expression in PSCs through NF-κB (Fig. 5f, g).

### NF-κB regulated ESE3 expression by directly binding to its promoter region

NF-κB regulates target gene expression through binding to the promoter and enhancer regions containing κB consensus sequences 5′-GGGRNWYYCC-3′ (N, any base; R, purine; W, adenine or thymine; Y, pyrimidine) [34]. We identified a κB-binding site in the promoter region of the human *ESE3* gene (Fig. 5h, top). ChIP assay was performed on ihPSCs to examine whether NF-κB (p65) directly binds to the ESE3 promoter. The fragment immunoprecipitated by anti-p65 antibody was detected in the chromatin fractions pulled down by anti-p65 antibody (Fig. 5h, bottom). We constructed a full-length ESE3 luciferase promoter vector and co-transfected this reporter construct with or without siNF-κB (p65) into HEK293 cell and ihPSCs to determine whether the binding of NF-κB (p65) activates ESE3 promoter. Luciferase analysis showed that the suppressed NF-κB activity by siNF-κB (p65) remarkably decreased the ESE3 promoter activity in HEK293 cells and ihPSCs (Fig. 5i). This κB-binding site was mutated to determine whether the κB-binding site is required for NF-κB to transactivate the ESE3 promoter. As shown in Fig. 5i, the mutation of the κB-binding site almost abolished the transactivation of the ESE3 promoter by NF-κB.

### IL-1β/ESE3 (PSCs)/IL-1β-positive feedback loop promoted PDAC progression

**Fig. 6:**
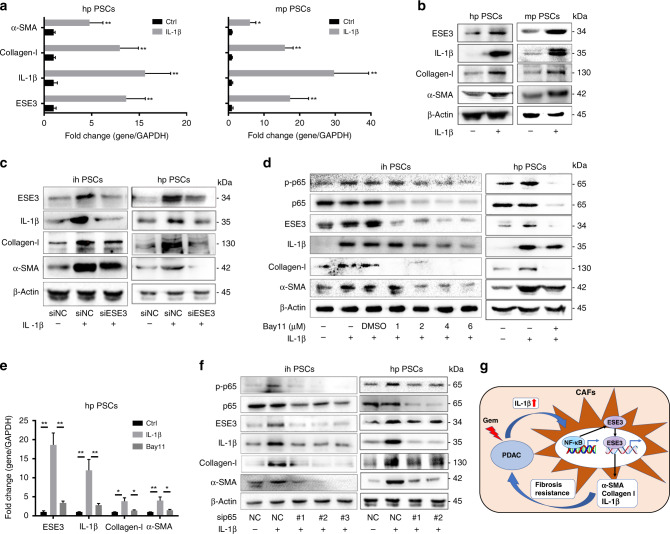
IL-1β/ESE3 (PSCs)/IL-1β-positive feedback loop promoted PDAC progression. mRNA (**a**) and protein (**b**) expression levels of ESE3, α-SMA, collagen-I and IL-1β in hpPSCs and mpPSCs stimulated with recombinant human IL-1β (100 ng/mL, 24 h). **c** Western blot analyses of ESE3, α-SMA, collagen-I and IL-1β expression in ihPSCs and hpPSCs with IL-1β stimulation and/or knockdown of ESE3 expression. Western blot (**d**) and RT-PCR (**e**) analyses of p-p65, ESE3, α-SMA, collagen-I and IL-1β expression in ihPSCs and hpPSCs with IL-1β stimulation and/or NF-κB (p65) inhibitor Bay11 treatment. **f** Western blot analyses of p-p65, ESE3, α-SMA, collagen-I and IL-1β expression in ihPSCs and hpPSCs with IL-1β stimulation and/or knockdown of NF-κB (p65) expression. **g** Schematic of the IL-1β/NF-κB/ESE3 signalling axis that increased α-SMA, collagen-I and IL-1β expression in PSCs and promoted fibrosis and chemoresistance. *P < 0.05 and **P < 0.01.

As shown in Fig. 6a, b, the mRNA and protein expression of ESE3, α-SMA, collagen-I and IL-1β was induced by recombinant IL-1β, which is consistent with the activation of PSCs by IL-1β. Blocking experiments were conducted using PSCs and PSCs-siESE3 to further understand the role of ESE3 in IL-1β-mediated PDAC progression. After IL-1β stimulation, the expression levels of α-SMA, collagen-I and IL-1β in PSCs were remarkably upregulated. Conversely, the expression levels of α-SMA, collagen-I and IL-1β were downregulated in PSC-siESE3 cell lines compared with the PSC-siNC group with IL-1β stimulation (Fig. 6c). Furthermore, we tested the role of NF-κB (p65) in the IL-1β-induced cascade using Bay11. When NF-κB (p65) activity was inhibited by Bay11, the protein and mRNA expression levels of ESE3, α-SMA, collagen-I and IL-1β were reduced in ihPSCs and hpPSCs (Fig. 6d, e). Moreover, sip65 was used to suppress NF-κB (p65) activity and obtained the same result as Bay11 for blocking (Fig. 6f). Thus, the IL-1β/ESE3 (PSCs)/α-SMA, collagen-I and IL-1β-positive feedback loop promoted PDAC progression (Fig. 6g).

## Discussion

Pancreatic cancer is the seventh leading cause of cancer deaths worldwide. Although the past few years have seen improvements in first-line and second-line palliative therapies, the 5-year survival rate of PDAC remains poor [35].

ESE3 is a member of the highly diverse ETS superfamily. Our group previously reported that tumoural ESE3 is a tumour-suppressing transcription factor that directly inhibits PDAC metastasis by upregulating E-cadherin [18]. In addition, tumoural ESE3 inhibits the expression of TGFβ1 and GM-CSF to promote the immune-suppressive TME in PDAC [36].

Previous research suggested that the function of ESE3 is context-dependent [37, 38]. Tugores et al. reported that ESE3 is a downstream signal of the MAPK pathway [39]. ESE3 plays a critical role in tumour cells and is also expressing in mast cells and monocytes. Furthermore, the mRNA and protein expression of ESE3 can be induced by IL-1β or TNF-α in human bronchial smooth muscle cells and fibroblasts by the MAPK pathway [25]. In this study, ESE3 was also expressed in PSCs and can be upregulated by IL-1β. ESE3 (PSCs) promotes PDAC fibrosis, proliferation, chemoresistance and poor prognosis. The result seems paradox with the function of ESE3 (PDACs) in reported literature, but the discrepancy may be due to ESE3 expression in different cell types and TME, which needs further exploration. *ESE3* as an oncogene promotes thyroid tumourigenesis [22], ovarian cancer [23] and gastric cancer [24] progression.

IL-1β plays a key role in carcinogenesis and tumour growth owing to its importance in mediating the inflammatory response. Increased IL-1β levels in body fluids are correlated with worse cancer prognosis, chemoresistance and invasiveness [12]. IL-1β promotes chronic inflammation, fibrosis, endothelial cell activation, tumour angiogenesis and the induction of immunosuppressive cells to promote tumour progression [4, 11, 15, 40]. Previous studies posit that IL-1β in the PDAC microenvironment is produced by the stroma [4, 32, 33]. Recent research found that PDAC cell-derived IL-1β also plays a critical role in promoting PSC activation and tumour progression [13]. Our data confirmed that IL-1β can be expressed in PDAC cell lines, and the secreted IL-1β in tumour CM was remarkably upregulated after Gem treatment, which may be the reason for the secondary chemoresistance. Our data demonstrated that PDAC cell-derived IL-1β can induce ESE3 overexpression in PSCs by activating the NF-κB signal pathway. NF-κB (p65) directly binds to the ESE3 promoter and activates its expression. Silverman reported that IL-1β induces ESE3 mRNA expression by activating the MAPK pathway in bronchial smooth muscle cells and fibroblasts [25]. Our results uncovered a new mechanism of IL-1β in inducing ESE3 expression through the NF-κB signal pathway in PSCs.

In vitro experiments using cell lines demonstrated that the CM from ESE3-overexpressed/knockdown PSCs influenced PDAC proliferation and chemoresistance. Experiments in the vivo mouse model confirmed the function of ESE3 (PSCs) in promoting tumour growth, chemoresistance and fibrosis. Furthermore, our data demonstrated that the mRNA and protein levels of α-SMA, collagen-I and IL-1β were remarkably upregulated by ESE3. Mechanically, ESE3 directly binds to the promoter regions of α-SMA, collagen-I and IL-1β and activates their expression. α-SMA, collagen-I and IL-1β overexpression is the prominent feature of PSC activation [41, 42]. PDAC cell- and PSC-derived IL-1β induces ESE3 overexpression to promote PSC activation and the upregulation of α-SMA, collagen-I and IL-1β. Importantly, clinical data suggested that ESE3 (PSC) expression was positively correlated with pTNM, tumour size, CA19-9, CEA and CA242. ESE3 overexpression in PSCs was an independent risk factor for OS and DFS amongst patients with PDAC.

Together, these data demonstrated a critical IL-1β/ESE3(PSCs)/IL-1β positive feedback loop that drives fibrosis, chemoresistance and PDAC progression. Inhibiting this positive feedback loop might be a novel strategy to reduce tumour fibrosis and increase chemotherapeutic efficacy in PDAC.

## Supplementary information

Supplementary Tables

Figure S1

Figure S2

Figure S3

Supplementary Figure Legends
