# Supplementary material for: ESE3-positive PSCs drive pancreatic cancer fibrosis, chemoresistance and poor prognosis via tumour–stromal IL-1β/NF–κB/ESE3 signalling axis
Source: Br J Cancer. 2022 Aug 19;127(8):1461–72. doi: 10.1038/s41416-022-01927-y (PMC9553871; doi:10.1038/s41416-022-01927-y)
Supplement: Supplementary file 1 — Supplementary Tables [file 41416_2022_1927_MOESM1_ESM.docx]

**Table S1. Clinicopathologic characteristics of PDAC patients**

| **Parameters** | **Cases (n)** | **(%)** |
| --- | --- | --- |
| Gender |  |  |
| Male | 59 | 55.14 |
| Female | 48 | 44.86 |
| Age (years) |  |  |
| <65 | 78 | 72.90 |
| ≥65 | 29 | 27.10 |
| Differentiation |  |  |
| Well/ Moderate | 83 | 77.57 |
| Poor | 24 | 22.43 |
| pTNM |  |  |
| I-II | 30 | 28.04 |
| III | 77 | 71.96 |
| Tumor size (cm) |  |  |
| <5 | 59 | 55.14 |
| ≥5 | 48 | 44.86 |
| LN metastasis |  |  |
| N0 | 27 | 25.23 |
| N1 | 80 | 74.77 |
| Histological grade |  |  |
| G1 | 76 | 71.03 |
| G2, G3 | 31 | 28.97 |
| CA19-9 (U/mL) |  |  |
| <40 | 32 | 29.91 |
| ≥40 | 75 | 70.09 |
| CEA (μg/L) |  |  |
| <5 | 67 | 62.62 |
| ≥5 | 40 | 37.38 |
| CA242 (IU/mL) |  |  |
| <20 | 46 | 42.99 |
| ≥20 | 61 | 57.01 |

**Table S2. Antibodies and primers**

| **Antibodies** | | | | | |
| --- | --- | --- | --- | --- | --- |
| **Name** | **Manufacturer** | **Number** | | **Type** | **Usage** |
| ESE3 | LifeSpan BioSciences | LS-B11884 | | Polyclonal | WB |
|  | Abcam | ab105375 | | Polyclonal | IHC-P |
|  | Abcam | ab220113 | | Polyclonal | ChIP, IF |
| NF-κB p65(D14E12) | Cell Signaling Technology | #8242 | | Monoclonal | WB, ChIP |
| Phospho-NF-κB -p65(Ser536)(93H1) | Cell Signaling Technology | #3033 | | Monoclonal | WB |
| α-SMA | Sigma-Aldrich | A5228 | | Monoclonal | WB, IHC-P, IF |
| Collagen I | Abcam | Ab34710 | | Polyclonal | WB, IHC-P |
| IL1β | Proteintech | 16806-1-AP | | Polyclonal | WB, IHC-P |
| Lamin B1 | Proteintech | 66095-1-lg | | Monoclonal | WB |
| α-tublin | Proteintech | 11224-1-AP | | Monoclonal | WB |
| β-actin | Proteintech | 60008-1-lg | | Monoclonal | WB |
| CK-19 | Abcam | ab7755 | | Monoclonal | IF |
| Ki-67 | Abcam | ab16667 | | Monoclonal | IHC |
| DAPI | Beyotine Biotechnology | C1002 | |  | IF |
| **shRNA** | **sequences (5’-3’)** |  | |  |  |
| Human ESE3 | CCGGGCCAATTGTATCCCTTTCCAACTCGAGTTGGAAAGGGATACAATTGGCTTTTT | | | | |
| Murine ESE3 | AAAAGCAACCTACAGCATCTCAAGTTTGGATCCAAACTTGAGATGCTGTAGGTTGC | | | | |
| **siRNAs** | **sequences (5’-3’)** | | | | |
| ESE3-homo-309 | GCCAGUGGCAUGAAAUUCATT/ UGAAUUUCAUGCCACUGGCTT | | | | |
| ESE3-homo-979 | CAGCCGAGCUAUGAGAUAUTT/ AUAUCUCAUAGCUCGGCUGTT | | | | |
| ESE3-homo-1032 | GACGAAGACUGGUAUAUAATT/ UUAUAUACCAGUCUUCGUCTT | | | | |
| ESE3-mus-284 | CCUCAGUACUGGACCAAAUTT/ AUUUGGUCCAGUACUGAGGTT | | | | |
| ESE3-mus-451 | GCUGCUCUACAGCAACCUATT/ UAGGUUGCUGUAGAGCAGCTT | | | | |
| ESE3-mus-1010 | GGGAAGAAUGCUCGUGGAUTT/ AUCCACGAGCAUUCUUCCCTT | | | | |
| NF-κB-homo #1 | AAGAAGAAGUGCAGAGGAATT / UUCCUCUGCACUUCUUCUUTT | | | | |
| NF-κB-homo #2 | UGACAAAAGUGAUGACAAATT / UUUGUCAUCACUUUUGUCATT | | | | |
| NF-κB-homo #3 | CUGGAUGACUCUUGGGAAATT / UUUCCCAAGAGUCAUCCAGTT | | | | |
| Positive control | UGACCUCAACUACAUGGUUTT / AACCAUGUAGUUGAGGUCATT | | | | |
| Negative control | UUCUCCGAACGUGUCACGUTT / ACGUGACACGUUCGGAGAATT | | | | |
| **Primer sequences (5’-3’)** | | | | | |
| qPCR ESE3-homo | F: GGGCTCAGATCTCCATGACA | | R: ATCCTGGGTTCTTGTCTGGG | | |
| qPCR ESE3-mus | F: ATGCAATGTTTCCAGCGGTT | | R: AACTCCTGCAGACTCATGCT | | |
| qPCR NF-κB-homo | F: AGTGGCAACGGGTAAACTTG | | R: GCTTCACAGGACCAGACACA | | |
| qPCR NF-κB-mus | F: CTGACCTGAGCCTTCTGGAC | | R: GCAGGCTATTGCTCATCACA | | |
| qPCR Collagen I-homo | F: ATGTGCCACTCTGACTGGAA | | R: CTTGTCCTTGGGGTTCTTGC | | |
| qPCR Collagen I-mus | F: TGAACGTGACCAAAAACCAA | | R: GCAGAAAAGGCAGCATTAGG | | |
| qPCR α-SMA-homo | F: ACCCAGCACCATGAAGATCA | | R: TTTGCGGTGGACAATGGAAG | | |
| qPCR α-SMA-mus | F: CTGACAGAGGCACCACTGAA | | R: CATCTCCAGAGTCCAGCACA | | |
| qPCR IL1β-homo | F: GGAGAATGACCTGAGCACCT | | R: GGAGGTGGAGAGCTTTCAGT | | |
| qPCR IL1β-mus | F: GCCCATCCTCTGTGACTCAT | | R: AGGCCACAGGTATTTTGTCG | | |
| qPCR GAPDH-homo | F: GGAGCGAGATCCCTCCAAAAT | | R: GGCTGTTGTCATACTTCTCATGG | | |
| qPCR GAPDH-mus | F: AGCAGTCCCGTACACTGGCAAAC | | R: TCTGTGGTGATGTAAATGTCCTCT | | |
| **ChIP** |  | | | | |
| ESE3-Collagen I EBS | CTCCCCACTCCATCTCTCAA / GTGCAGGCAAGGGAGTTTTA | | | | |
| ESE3-IL1β EBS1 | TCCTGCAATTGACAGCAGAGAGC / GGGTACAATGAAGGGCCAAT | | | | |
| ESE3-IL1β EBS2 | CGGCAGGTGCCTGTAGTC / GAGTGCAGTGGCACGATCT | | | | |
| ESE3-α-SMA EBS1 | CACTCAGGCAGCGACTTACA / TGAGCTTCTGGAGGCTTGTT | | | | |
| ESE3-α-SMA EBS2 | AGAGTGTGGCCTGAGGTTGT / GCTGAGATGTCTGGGTCCAT | | | | |
| ESE3-α-SMA EBS3 | TTGTCAACTTTTTGGGATTTCA / CCATCCTTAACCCCTTTTGC | | | | |
| NF-κB-ESE3 NBS | GAGCCAGTGTTTGTAATCCTT / AGGATGTTAAGCAATAAGCCAC | | | | |

| **Table S3. Association between ESE3 expression in PSCs and clinicopathological feature of patients with PDAC tissues** | | | | | |
| --- | --- | --- | --- | --- | --- |
|  | Total | ESE3 expression | | χ^2^ | *P* value |
|  |  | High/Moderate | Low/absent |  |  |
| Gender |  |  |  | 1.551 | 0.213 |
| Male | 59 | 39 | 20 |  |  |
| Female | 48 | 37 | 11 |  |  |
| Age (year) |  |  |  | 0.587 | 0.444 |
| <65 | 78 | 57 | 21 |  |  |
| ≥65 | 29 | 19 | 10 |  |  |
| Differentiation |  |  |  | 0.001 | 0.981 |
| Well/Moderate | 83 | 59 | 24 |  |  |
| Poor | 24 | 17 | 7 |  |  |
| pTNM |  |  |  | 6.343 | 0.012* |
| I-II | 30 | 16 | 14 |  |  |
| III | 77 | 60 | 17 |  |  |
| Tumor size (cm) |  |  |  | 6.405 | 0.011* |
| <5 | 59 | 36 | 23 |  |  |
| ≥5 | 48 | 40 | 8 |  |  |
| LN metastasis |  |  |  | 0.163 | 0.687 |
| - | 27 | 20 | 7 |  |  |
| + | 80 | 56 | 24 |  |  |
| Histological grade |  |  |  | 0.213 | 0.645 |
| G1 | 76 | 53 | 23 |  |  |
| G2 and G3 | 31 | 23 | 8 |  |  |
| CA19-9 (U/mL) |  |  |  |  |  |
| <40 | 32 | 18 | 14 | 4.845 | 0.028* |
| ≥40 | 75 | 58 | 17 |  |  |
| CEA (μg/L) |  |  |  |  |  |
| <5 | 67 | 42 | 25 | 6.060 | 0.014* |
| ≥5 | 40 | 34 | 6 |  |  |
| CA242 (IU/mL) |  |  |  |  |  |
| <20 | 46 | 28 | 18 | 4.046 | 0.044* |
| ≥20 | 61 | 48 | 13 |  |  |
| Abbreviation: LN, lymph node. **P*<0.05(Chi-Square Test) | | | | | |

| **Table S4. Univariate and multivariate analysis** **of clinicopathological factors for OS and DFS** | | | | | | | | | | |
| --- | --- | --- | --- | --- | --- | --- | --- | --- | --- | --- |
| **Univariate analysis** | | | |  | | | |  | | |
| Parameters | | adverse/  advantage | | OS | | | | DFS | | |
|  |  |  |  | HR (95%CI)^a^ | | *P^b^* | | HR (95%CI) | | *P* |
| ESE3 | | high/low | | 1.92 (1.07,3.44) | | 0.030^*^ | | 1.89 (1.02,3.52) | | 0.044^*^ |
| Gender | | man/woman | | 0.93 (0.57,1.54) | | 0.785 | | 0.69 (0.41,1.17) | | 0.171 |
| Age(year) | | ≥65/＜65 | | 0.99 (0.97,1.01) | | 0.386 | | 0.99 (1.00,1.02) | | 0.434 |
| Differentiation | | poor/ well | | 0.64 (0.29,1.42) | | 0.271 | | 0.59 (0.25,1.39) | | 0.224 |
| Tumor size(cm) | | ≥5/＜5 | | 1.66 (1.01,2.72) | | 0.046^*^ | | 1.80 (1.08,3.01) | | 0.024^*^ |
| LN metastasis | | +/- | | 1.03 (0.59,1.82) | | 0.910 | | 1.45 (0.77,2.73) | | 0.254 |
| CA19-9 | | +/- | | 1.000 (1.000,1.001) | | 0.283 | | 1.000 (0.999,1.001) | | 0.944 |
| CEA | | +/- | | 1.026 (1.002,1.050) | | 0.031^*^ | | 1.027 (1.002,1.053) | | 0.034^*^ |
| CA242 | | +/- | | 1.000 (0.998,1.003) | | 0.601 | | 1.000 (0.997,1.003) | | 0.899 |
| **Multivariate analysis** | | | | | | | | | | |
| Parameters | adverse | | OS | | | | DFS | | | |
|  |  |  | HR (95%CI)^a^ | | *P^b^* | | HR (95%CI) | | *P* | |
| ESE3^c^ | ESE3 high | | 1.92 (1.07,3.44) | | 0.030^*^ | | 1.89 (1.02,3.52) | | 0.044^*^ | |
| Model 1^d^ | ESE3 high | | 3.42 (1.63,7.19) | | 0.001^*^ | | 1.92 (1.04,3.57) | | 0.039^*^ | |
| Model 2^e^ | ESE3 high | | 2.35 (1.11,4.94) | | 0.025^*^ | | 2.65 (1.11,6.32) | | 0.028^*^ | |
| Model 3^f^ | ESE3 high | | 2.65 (1.31,5.39) | | 0.007^*^ | | 3.87 (1.70,8.82) | | 0.001^*^ | |
| ^a^ HR,hazard ratio; CI, confidence interval. (all such values). | | | | | | | | | | |
| ^b^ Analysis by Cox proportional hazards model. | | | | | | | | | | |
| ^c^ The crude model. (Without the variable adjustment model). | | | | | | | | | | |
| ^d^ Model 1 adjusted for age and gender. | | | | | | | | | | |
| ^e^ Model 2 additionally adjusted differentiation, tumor size, lymph node metastasis, pTNM. | | | | | | | | | | |
| ^f^ Model 3 additionally adjusted pancreatic tumor biomarkers. | | | | | | | | | | |
