# Supplementary figures and images for: ESE3-positive PSCs drive pancreatic cancer fibrosis, chemoresistance and poor prognosis via tumour–stromal IL-1β/NF–κB/ESE3 signalling axis
Source: Br J Cancer. 2022 Aug 19;127(8):1461–72. doi: 10.1038/s41416-022-01927-y (PMC9553871; doi:10.1038/s41416-022-01927-y)

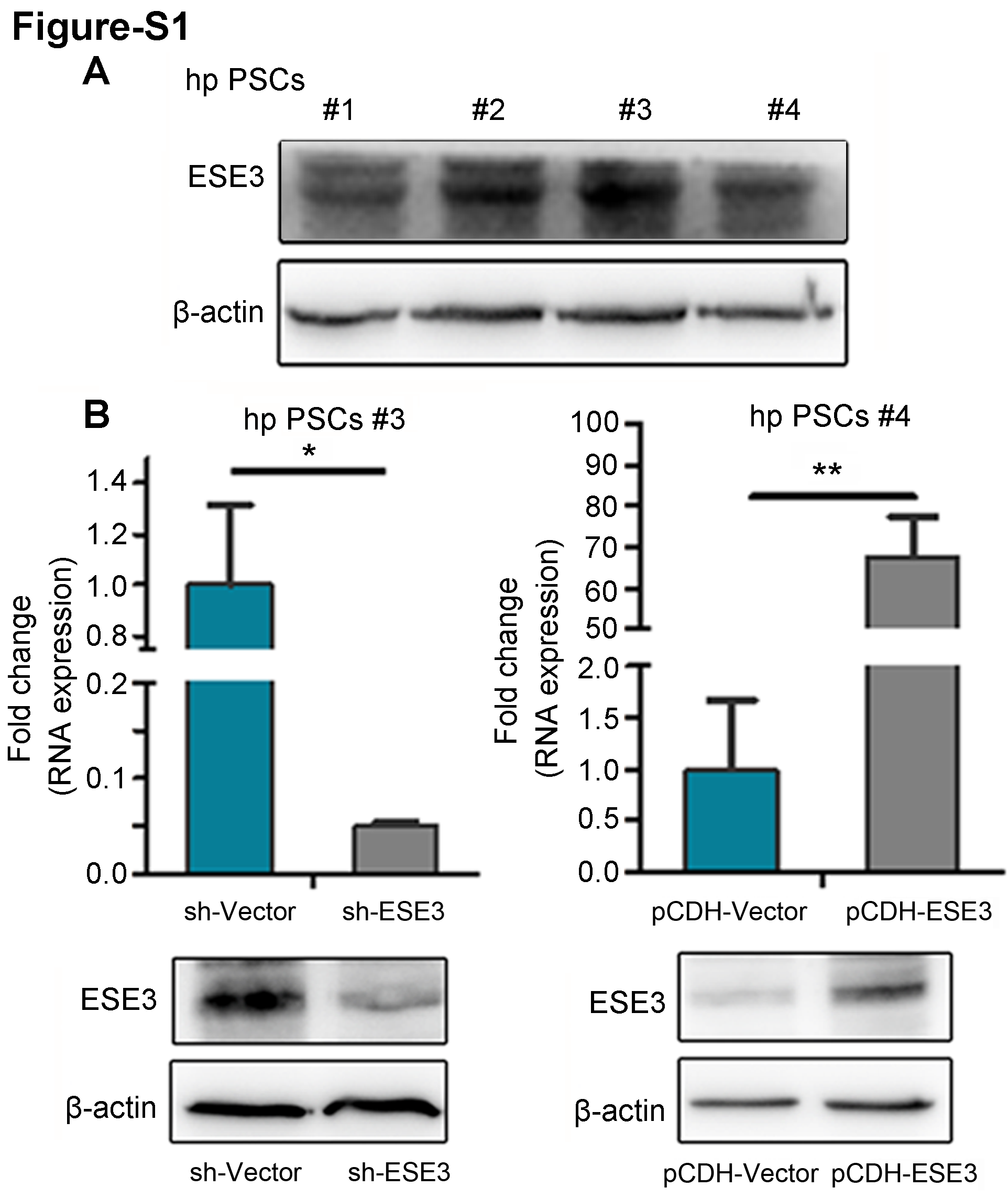

Supplement: Supplementary file 2 — Figure S1 [file 41416_2022_1927_MOESM2_ESM.tif]

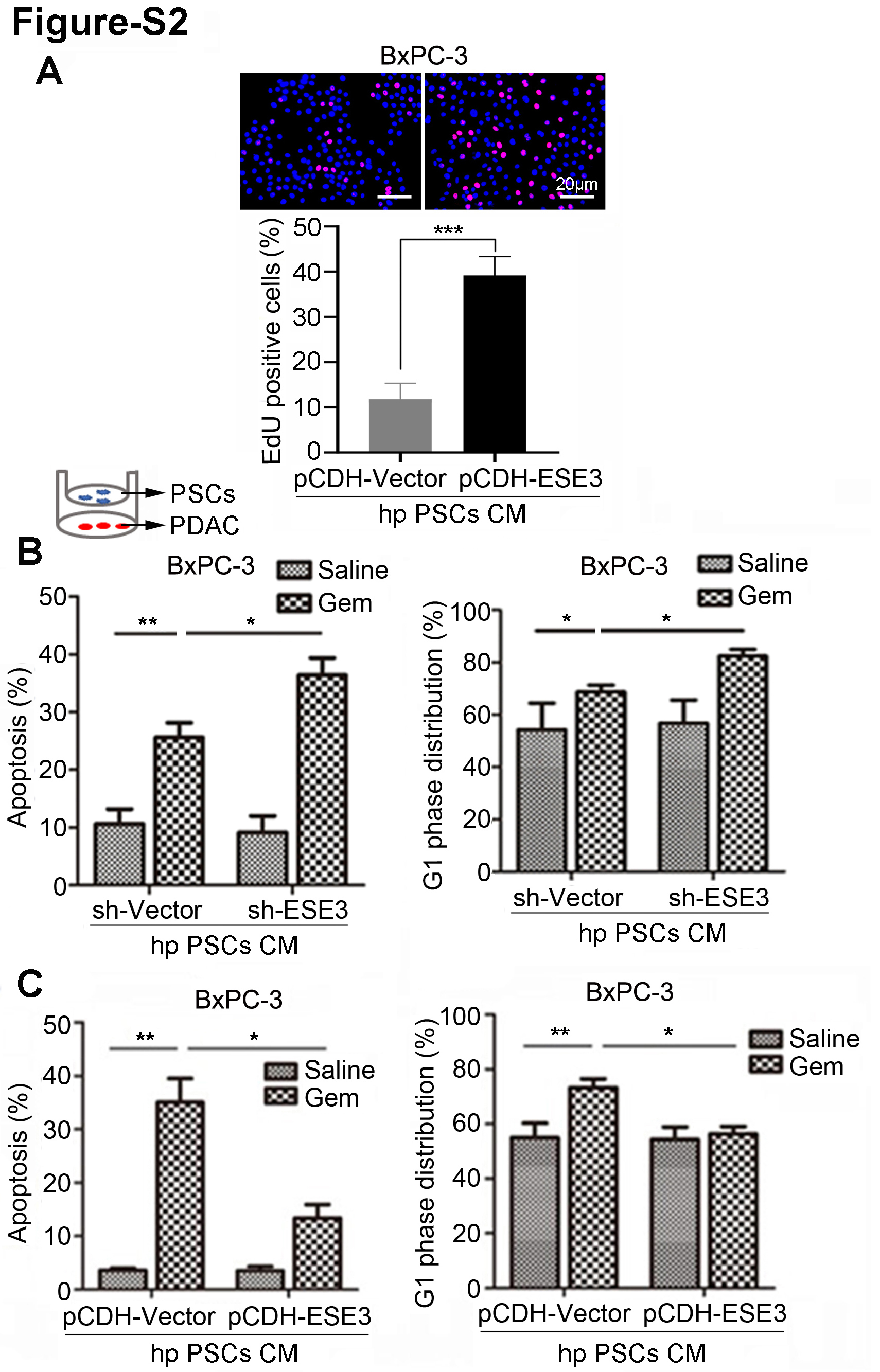

Supplement: Supplementary file 3 — Figure S2 [file 41416_2022_1927_MOESM3_ESM.jpg]

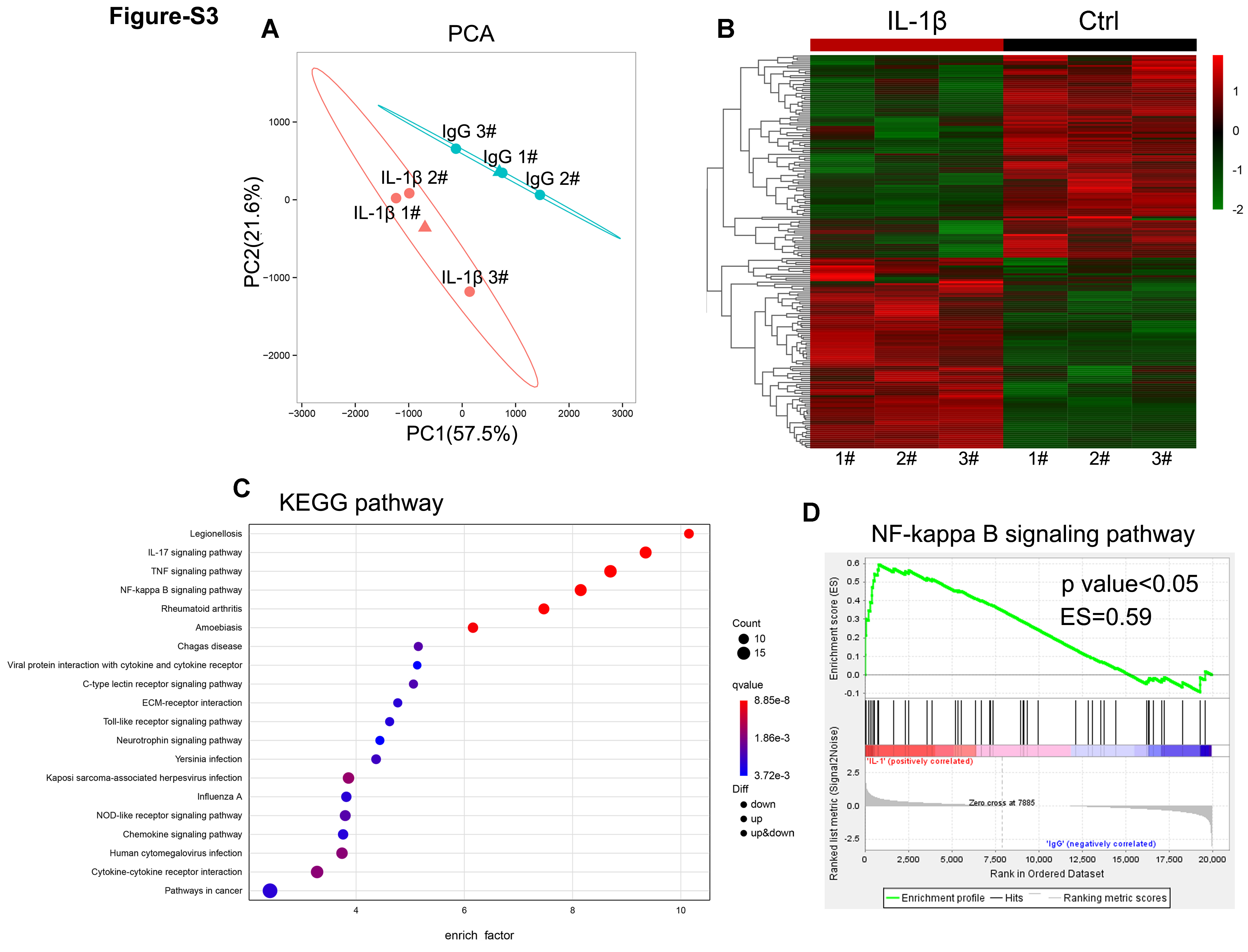

Supplement: Supplementary file 4 — Figure S3 [file 41416_2022_1927_MOESM4_ESM.jpg]
