# Supplementary material for: ESE3-positive PSCs drive pancreatic cancer fibrosis, chemoresistance and poor prognosis via tumour–stromal IL-1β/NF–κB/ESE3 signalling axis
Source: Br J Cancer. 2022 Aug 19;127(8):1461–72. doi: 10.1038/s41416-022-01927-y (PMC9553871; doi:10.1038/s41416-022-01927-y)
Supplement: Supplementary file 5 — Supplementary Figure Legends [file 41416_2022_1927_MOESM5_ESM.docx]

**Supplementary Figure Legends**

**Figure S1**. **(A)** Measurement the ESE3 expression in four human primary PSCs by the Western-blot assay. **(B)** RT-PCR and Western-blot experiment verification ESE3 mRNA and protein expression in PSCs transfected with sh-ESE3/ sh-Vector and pCDH-ESE3/ pCDH-Vector plasmid.

**Figure S2**. (**A**) EdU experiment evaluation of the proliferation of BxPC-3 cell line cultured with the CM of pCDH-ESE3/pCDH-Vector hpPSCs for 48 h. Flow cytometry was performed to analyse the apoptosis and G1 phase distribution of BxPC-3 cell line indirectly co-cultured with sh-ESE3/sh-Vector hpPSCs **(B)** or pCDH-ESE3/pCDH-Vector hpPSCs **(C)** and then treated with Gem (2 μM) for 24 h. **P* < 0.05 and ***P* < 0.01.

**Figure S3**. (A) PCA analysis of three biological replicates in IL-1β and control treatment group. (B) Heatmap of DEG between IL-1β and control treatment group. (C) KEGG enrichment analysis of positively correlated pathways in IL-1β treatment group. (D) GSEA enrichment analysis of NF-kappa B signaling pathway between IL-1β and control treatment group.

.
